# *Phthorimaea absoluta* in potato cultivars: tolerance analysis and implications for integrated pest management

**DOI:** 10.1007/s41348-025-01157-3

**Published:** 2025-09-18

**Authors:** Lindiwe Mahlangu, Khayalethu Ntushelo, Phumzile Sibisi

**Affiliations:** Department of Agriculture and Animal Health, University of South Africa, Private Bag X6, Johannesburg, 1710 South Africa

**Keywords:** Gas exchange parameters, Genetic resistance, Integrated pest management, Nutrient assimilation, *Phthorimaea absoluta*, Potato cultivar tolerance

## Abstract

*Phthorimaea absoluta* is an invasive pest that causes substantial damage to Solanaceous crops, such as tomato and potato. This study evaluated the resistance of five commonly cultivated potato varieties to *P. absoluta* infestation in field conditions across South Africa. Morphological parameters, gas exchange, and nutrient assimilation parameters were analysed in both infested and uninfested plants. Valor exhibited the highest tolerance, showing minimal leaf damage, fewer mines, optimal gas exchange, and greater assimilation of key nutrients (e.g. iron, zinc, and manganese), which may enhance its defensive capacity against pests. Conversely, Sifra was identified as the most susceptible cultivar, with reduced nutrient uptake and significant physiological disruption in response to infestation. Infested plants showed a decrease in photosynthesis, transpiration, and stomatal conductance, along with an increase in intercellular CO_2_, highlighting the harmful effects of *P. absoluta*. These findings support the selection of cultivars in breeding and integrated pest management programmes, especially in African contexts where field data are limited.

## Introduction

Potato (*Solanum tuberosum* L.) is one the most significant food crops in the world (Sprenger et al. 2018). It is the most widely grown crop in South Africa with over 2,491,000 tonnes produced (Abstract of Agricultural Statistics 2022). The potato tuber is eaten in various forms, such as mash, fries, or chips. Since its domestication, the potato plant has been subject to pest and disease problems, ranging from local infestations to wide-scale epidemics that reduce production severely. In recent years, a severely damaging pest of tomato, namely *Phthorimaea absoluta* (Meyrick, 1917) (Lepidoptera: Gelechiidae), has been recorded on potato crops.

In 2016, *P. absoluta* invaded South Africa, causing significant yield losses in open fields and tunnel-grown tomatoes nationwide (Visser et al. 2017). *Phthorimaea absoluta* inflicts damage on host plants at any stage (European Plant Protection Organization (EPPO), 2005). The injuries caused by this pest are found on inflorescences, stems, and fruits (Arnó and Gabarra 2010). This damage led to a 100% loss of tomato crops (Chhetri 2018). In the absence of tomato, the preferred host plant of *P. absoluta* (Ajibade et al. 2017; Erdoğan and Babaroğlu 2014; Shiberu and Getu 2017; Uzun et al. 2015; Younes et al. 2018), the pest can cause economic damage to potato (Pereyra et al., 2006; Caparros et al. 2013; Mahlangu et al. 2022; Yang et al. 2024).

*Phthorimaea absoluta*, originally a pest of tomato, has been reported to attack potato crops, causing damage to leaves and stems (Sperdouli et al. 2021). Although the pest does not directly damage the tubers, it contributes to yield reduction. Pest infestations impact plant physiology, particularly gas exchange and nutrient assimilation (Galdino et al. 2015; Gómez-Trejo et al. 2021). *Phthorimaea absoluta* damages leaf tissues, which reduces chlorophyll levels and photosynthesis while disrupting gas exchange. This limits carbon assimilation and energy production. Furthermore, vascular damage impairs nutrient uptake, weakening the plant’s metabolism and defence, ultimately leading to reduced growth and yield (Mahlangu et al. 2022). These physiological processes are crucial for crop health and therefore disruptions can lead to impaired growth and reduced yields (Sperdouli et al. 2021). Nutrient assimilation is vital for how plants defend themselves against pest infestations (Trapet et al. 2021). Various methods of pest control exist, but integrating pest control measures is more sustainable using one measure.

Integrated pest management (IPM) is a comprehensive approach that combines different effective management strategies to control insect pests (Rwomushana et al. 2019). It integrates chemical control, biological control, crop rotation, effective monitoring, mass trapping, and resistant cultivars (Mansour et al. 2018; Rwomushana et al. 2019), among other interventions. IPM has been proven successful in controlling many insect pests (Ghaderi et al. 2017). By minimising the use of chemicals, IPM delays the build-up of pesticide resistance (Stenberg 2017) and considers the cost versus the benefit of farming, the sale, and consumption of produce, and the environment (Neves et al. 2006). This management approach is being adapted to control the destructive insect pest *P. absoluta* which affects Solanaceae crops (Biondi et al. 2018; Uzun et al. 2015). As part of a viable IPM strategy, cultivar resistance offers tremendous benefits to farmers. For example, in tomato production, the combination of pheromone traps and resistant cultivars has significantly reduced *P. absoluta* populations and delayed the development of chemical resistance (Desneux et al. 2022). Given that potatoes are produced in sixteen different climatic regions across South Africa (Potato South Africa (PSA), 2017), identifying resistant cultivars is crucial for sustainable production, particularly as the pest’s persistence threatens long-term yields.

Limited studies have addressed the impact of *P. absoluta* on crop growth, yield, gas exchange aspects and nutrient assimilation in potatoes, highlighting a gap in our understanding of host–pest interactions in this crop. While several studies have identified resistance mechanisms in tomato and potato cultivars in laboratory settings, such as the expression of insecticidal genes (Caparros et al. 2013; Tekinsoy et al. 2024), field-based evaluations of potato cultivar tolerance to *P. absoluta* are critically scarce, especially in African agricultural systems where environmental conditions and pest pressures differ significantly from those in other regions. The approach taken for the current work was based on the assumption that the relative tolerance or resistance of potato cultivars to *P. absoluta* was characterised by limiting the impact of the pest on growth, yield, physiological function, and nutrient assimilation.

The study aimed to assess the responses of each cultivar, including growth parameters, gas exchange, and nutrient assimilation patterns, to identify tolerant genotypes for integration into breeding programmes and IPM strategies. The primary objective was to evaluate the tolerance of five commercially important potato cultivars to *P. absoluta* infestations in field conditions in South Africa.

This study is among the first to evaluate field-grown potato cultivars under natural *P. absoluta* pressure in South Africa, providing region-specific insights for crop protection.

## Materials and methods

### Experimental site, conditions and research design

The study was conducted at the at the Horticulture Research Centre on the Science Campus of the University of South Africa in Florida, South Africa, with a daytime temperature of 18 to 28 °C and a night-time temperature 15 to and 18 °C during summer. The experimental plants were kept inside 60 × 60 × 90 cm net mesh cages and maintained outdoors in a completely randomised block design, with manual irrigation. Even though there was no established volume, the plants were irrigated as needed based on observed soil moisture to maintain similar levels of soil moisture among treatments and cultivars.

Five potato cultivars (Panamera, Mondial, Tyson, Sifra, and Valor), which are widely grown in South Africa, were selected for the study. The seeds were cultivated directly in potting soil in 20-cm experiential pots. At vegetative growth (four leaf compounds), the plants were infested with eight L1 *P. absoluta* instar larvae, and the other eight plants remained uninfested (Mahlangu et al. 2022). Although infestation intensity per unit leaf area was not calculated, a consistent number of larvae was applied to each plant to ensure uniform infestation pressure across treatments. The larvae used to establish the colony were initially obtained from the Agricultural Research Council’s—Vegetable and Ornamental Plants Institute (ARC-VOPI). Once the larvae had developed into adults, healthy tomato plants were introduced into the cage to allow the adults to lay eggs. After the eggs hatched, first instar (L1) larvae were used to conduct the experiments. Each cultivar was allocated 16 plants, eight of which were infested with the pest providing sufficient replication for statistical robustness. All the morphological characteristics and gas exchange measurements were conducted on each plant (n = 8 per treatment per cultivar), while nutrient assimilation was assessed using three replicates (n = 3).

### Data collection

#### Screening of susceptible and resistant cultivars

Plant height was measured using a measuring tape. After harvesting, potato tubers were collected, counted, recorded, and weighed using a scale in grams. The number of leaves per plant and plant scoring (including the number of mines per plant, the number of leaves damaged, and overall plant damage) were visually noted and recorded. The plants were scored at seven-day intervals from infestation following the protocol of Resende et al. (2006).

The scoring procedure of Resende et al. (2006) has the following scoring ranks: Number of mines per plant: 0 = no mines; 1 = small, rare mines; 2 = small- to medium-sized mines, rare, often towards the leaflet borders; 3 = medium- to large-sized mines, coalescent, deformed leaflet borders; 4 = large-sized lesions, coalescent, deformed leaflets; and 5 = whole leaflet surface damaged.

Number of leaves damaged: 0 = no leaves attacked; 1 = 0.1 to 5% leaves attacked; 2 = 5.1 to 20% leaves attacked; 3 = 20.1 to 50% leaves attacked; 4 = 50.1 to 80% leaves attacked; 5 = more than 80% leaves attacked.

Overall plant damage: 0 = no leaf damage; 1 = 0.1 to 5% of total leaf area damaged, small, non-coalescent lesions; 2 = 5.1 to 20% of total leaf area damaged, small- to medium-sized, non-coalescent lesions; 3 = 20.1 to 50% of leaf area damaged, medium- to large-sized lesions; 4 = 50 to 80.1% of leaf area damaged, numerous, large, coalescent lesions; 5 = more than 80.1% of leaf area damaged, completely deformed plants.

#### Gas exchange measurements

The gas exchange parameters were measured using a portable circuit and an infrared gas analysis system (Li-6800, Li-Cor Inc., Lincoln, NE, USA). The device was configured to measure assimilation rate; transpiration rate; ambient; intercellular CO_2_; stomatal conductance; and total conductance. One leaf was enclosed in a chamber to measure the light response curve. The measurements were done on two full-grown undamaged leaves per plant. The tank had a photosynthetic photon flux density of 1000 µmolm^−2^/s, a carbon dioxide saturation of 380 mol/mol, a gaseous movement of 500 µmol/s, and a temperature of 25 °C. The data were obtained between 8 and 10 a.m. The data were collected 35 days after infestation.

#### Nutrient assimilation

**Fig. 1:**
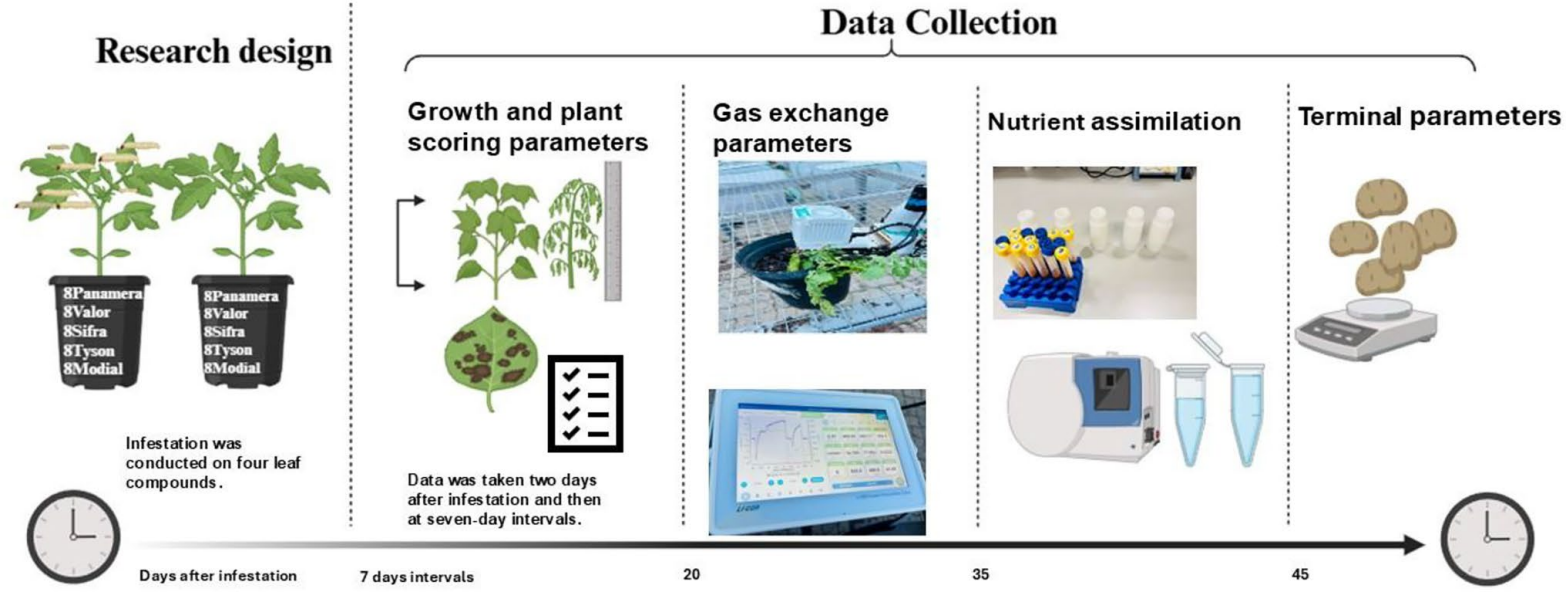
Illustrates the timeline, research design and data collection process for assessing plant responses to *Phthorimaea absoluta* infestation across various growth stages in five potato varieties (Panamera, Valor, Sifra, Tyson, and Mondial). Infestation was conducted on four leaf compounds, with the first data collected two days post-infestation and then at seven-day intervals up to 45 days. Key parameters measured included growth and plant scoring, gas exchange, nutrient assimilation, and terminal parameters

Nutrient assimilation was determined in potato leaves sampled 40 days after infestation. The samples were taken from leaves that showed little to no symptoms. The sampled leaves were crushed in liquid nitrogen using a mortar and pestle. Equal measures of samples weighing 0.5 g were mixed with 5 ml of 70% nitric acid and further processed using a temperature-controlled microwave digestion system fitted with a 20SVT50 rotor. The digestion procedure followed a five-step temperature programme, with each step maintained at 160 °C for 10 min, ensuring consistent fan cooling. The system operated in average temperature control mode, with the cooling temperature set at 70 °C and the cooling fan in operation. The digestion parameters were optimised, limiting power to 1800 W and setting the maximum temperature at 160 °C to prevent sample degradation. After digestion, the solution volumes were adjusted to 12 ml with distilled water, and the data were further analysed using inductively coupled plasma optical emission spectroscopy (ICP-OES) (Fig. 1).

### Data analysis

Morphological measurements (number of leaves per plant, plant height, tuber weight, and tuber quantity) were analysed using two-way ANOVA with a significance level of p < 0.05. The gas exchange parameters (assimilation, transpiration rate, ambient and intercellular CO_2_, stomatal conductance, and total conductance) and plant scoring (number of mines per plant, number of leaves damaged, and overall plant damage) were analysed using one-way ANOVA with a significance level of *p* < 0.05. Post hoc comparisons of means were conducted using Tukey’s Honestly Significant Difference (HSD) test. Tukey’s HSD was chosen for its robustness in controlling type I error across multiple comparisons in balanced designs. The nutrient assimilation between the most tolerant and most susceptible cultivars was analysed using an independent t test (*p* < 0.05). All the data were assessed for normality using the Shapiro–Wilk test, and homogeneity of variances was evaluated with Levene’s test before applying statistical analyses. The data were analysed using IBM SPSS Statistics 29. The measurement of morphological and gas exchange parameters was correlated by determining pairwise Pearson correlation using the JASP Team (2024) (JASP (Version 0.18.3) (computer software)).

## Results

The results of the study showed that *P. absoluta* inflicted varying levels of damage on the foliage of the five potato cultivars impacting morphological parameters, gas exchange, and nutrient assimilation. The cultivar Sifra was the most susceptible, and Valor was the least affected.

### Morphological parameters

Among the morphological parameters assessed were plant height, number of leaves per plant, number of mines per plant, leaf damage, and overall plant damage in response to pest feeding on the five cultivars studied. *Phthorimaea absoluta* significantly reduced and damaged the number of leaves resulting, in complete plant destruction in Sifra; Valor exhibited less damage, while the other cultivars showed intermediate effects.

**Fig. 2:**
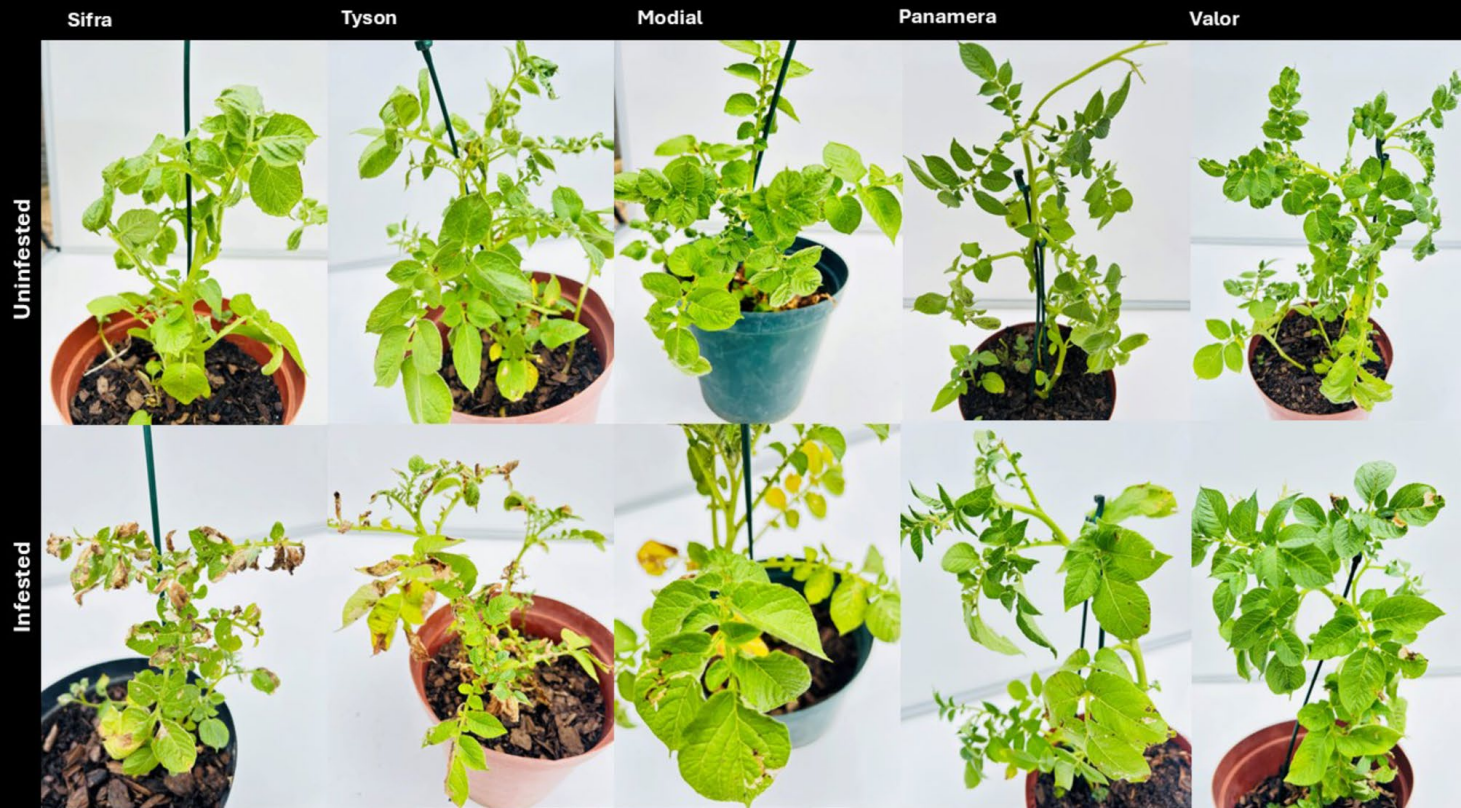
Potato cultivars in terms of their tolerance to susceptibility (from lowest to highest) 35 days after infestation

**Fig. 3:**
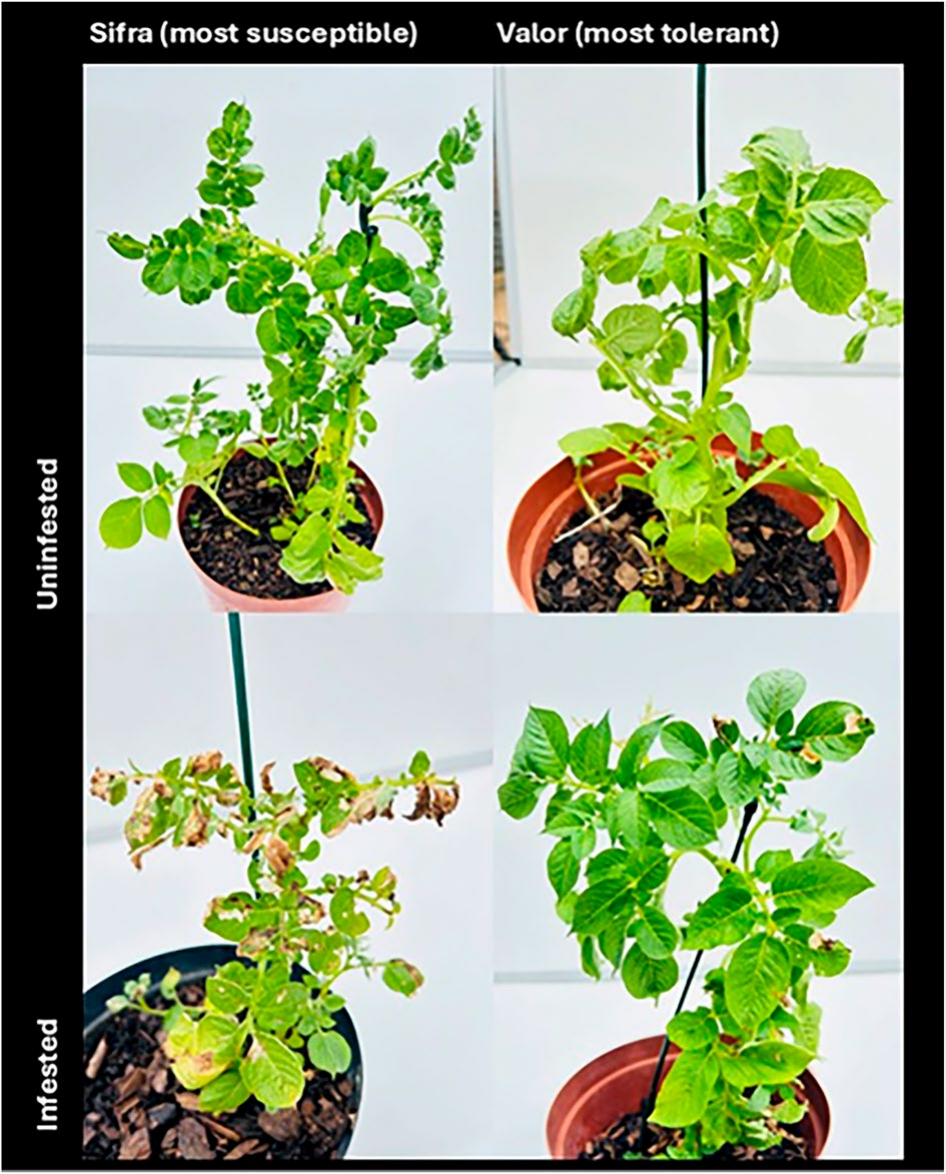
Plant damage on the most susceptible cultivar (Sifra) and most tolerant cultivar (Valor) 35 days after infestation

Statistically, the ranking order from most affected to least affected was Sifra, Tyson, Mondial, Panamera, and Valor (Figs. 2 and 3). Significant differences were found in the number of leaves per plant, plant height, tuber weight, number of mines per plant, number of damaged leaves, and overall plant damage.

Contrary to expectations, the infested plants were taller than the uninfested plants, except for Panamera. This may indicate a stress-induced elongation response, where plants prioritise vertical growth to escape or compensate for leaf damage. The plant height was ranked from shortest to tallest: Mondial, Sifra, Tyson, Panamera, and Valor, with a statistically significant difference (F (4, 70) = 3.44, *p* = 0.05). For leaf accumulation, uninfested plants developed more leaves than infested plants in all the cultivars except Panamera. Among infested plants, the leaf count ranked from lowest to highest was Mondial, Panamera, Valor, Sifra, and Tyson, with significant differences (F (4, 70) = 3.43, *p* < 0.001) (Table 1).

**Table 1:** Comparison of plant morphological parameters between uninfested and infested potato cultivars

	Cultivars	Infested (Low–high) Mean ± SD	Uninfested Mean ± SD	*P* = value
Number of leaves per plant				< 0.001
	Mondial^a^	68 ± 13.41	93.75 ± 20.98	
	Panamera^a^	76.75 ± 22.35	74.5 ± 12.03	
	Valor^b^	90.5 ± 15.44	109.125 ± 10.58	
	Sifra^bc^	96.87 ± 11.11	133.75 ± 17.62	
	Tyson^cd^	106.37 ± 9.16	127.25 ± 15.48	
Plant height (cm)				< 0.05
	Mondial^d^	21.14 ± 1.54	19.482 ± 2.09	
	Sifra^c^	21.37 ± 1.41	21.03 ± 0.88	
	Tyson^bc^	23.26 ± 1.00	21.89 ± 1.47	
	Panamera^a^	23.64 ± 3.49	25.64 ± 1.53	
	Valor^b^	25.64 ± 1.53	24.58 ± 1.60	
Tuber weight (g)				0.01
	Panamera^a^	2.8882 ± 0.32	3.7892 ± 0.45	
	Valor^c^	3.4952 ± 0.32	4.1773 ± 0.26	
	Mondial^abcd^	3.6015 ± 0.31	3.5686 ± 0.29	
	Sifra^bc^	3.6444 ± 0.39	4.0182 ± 0.62	
	Tyson^bcd^	3.9038 ± 0.29	4.0123 ± 0.41	
Tuber quantity				0.32
	Panamera	1.3461 ± 0.57	1.2556 ± 0.53	
	Mondial	1.4042 ± 0.30	1.1705 ± 0.42	
	Tyson	1.4664 ± 0.47	1.3824 ± 0.65	
	Valor	1.5495 ± 0.46	1.6509 ± 0.59	
	Sifra	1.6185 ± 0.18	2.0566 ± 0.34	

Means followed by different letters in columns indicate significant differences among treatments (Tukey’s test, *p* < 0.05)

When *P. absoluta* fed on the leaves of potatoes, it created superficial mines on the leaves. These superficial mines damaged and decreased the leaves on the plant, as well as the overall amount of foliage that could be produced by the plant. All the plant scoring methods were ranked from low to high: Valor, Panamera, Mondial, Tyson, and Sifra. Statistically significant differences were obtained in the number of mines per plant (F (4, 35) = 11.82, *p* < 0.001), number of leaves damaged (F (4, 35) = 7.20, *p* < 0.001), and overall plant damage (F (4, 35) = 15.56, *p* < 0.001). All the infested plants and cultivars displayed a higher number of superficial mines, damaged leaves, and overall plant damage (Table 2).

**Table 2:** Comparison of plant scoring parameters between different potato cultivars

	Cultivar (high–low)	Means ± SD	*P* = value
Number of mines per plant			< 0.001
	Sifra	15.63 ± 2.498^d^	
	Tyson	14.64 ± 1.969 ^cd^	
	Mondial	14.41 ± 4.447^bcd^	
	Panamera	9.37 ± 1.641^ab^	
	Valor	7.38 ± 2.08^a^	
Number of leaves damaged			< 0.001
	Sifra	1.79 ± 0.187^a^	
	Tyson	1.5 ± 0.076^ab^	
	Mondial	1.45 ± 0.29^ab^	
	Panamera	1.21 ± 0.606^a^	
	Valor	1 ± 0^a^	
Overall plant damage			< 0.001
	Sifra	1.63 ± 0.215^a^	
	Mondial	1.27 ± 0.194^b^	
	Panamera	1.14 ± 0.404^bc^	
	Valor	0.91 ± 0.152 ^cd^	
	Tyson	0.79 ± 0.076^d^	

Means followed by different letters in columns indicate significant differences among treatments (Tukey’s test < 0.05)

All the infested plants and cultivars exhibited increased superficial mines, damaged leaves, and overall plant damage, which impacted the potato tubers produced. The quantity of potato tubers, ranked from low to high, was Panamera, Mondial, Tyson, Valor, and Sifra. This ranking showed no statistically significant differences (F (4, 70) = 1.17, *p* = 0.32). Some infested plants demonstrated higher yields compared to the uninfested plants. However, the uninfested potatoes yielded large-size tubers. This disparity suggests that while stress from infestation may lead to an increased number of tubers, their development is impaired, resulting in smaller tubers and reduced overall weight. The weight of the infested tubers, ranked from low to high, was Panamera, Valor, Mondial, Sifra, and Tyson. The weight differences among the potatoes were statistically significant (F (4, 70) = 4.70, *p* = 0.01) (Table 1).

Sifra was indicated to be the most susceptible cultivar (Fig. 2). However, the infested plants did not produce the lowest number of leaves, tuber quantity and weight compared to other infested potato cultivars. Sifra showed the biggest difference between the uninfested and infested plants; hence, it had more mines, more leaves damaged, and more plant damage. Panamera and Valor were almost equally tolerant of *P. absoluta*. Valor had the least number of mines per plant, number of leaves damaged, and overall plant damage. Therefore, according to the plant scoring method, Valor is the most tolerant cultivar. The comparison of all cultivars is shown in Tables 1 and 2.

### Gas exchange parameters

The gas exchange parameters were measured across five cultivars: Panamera, Valor, Sifra, Tyson, and Mondial. The evaluated parameters included assimilation rate, transpiration rate, CO₂ concentrations, and conductance properties. Sifra was the most affected cultivar with a low assimilation rate, low transpiration rate, high ambient CO_2_ and high intercellular CO_2_. The remaining cultivars had varying responses on gas exchange parameters; they were ranked according to their responses (Table 3).

**Table 3:** Comparison of gas exchange parameters between different potato cultivars

	Cultivar (low–high)	Means ± SD	*P* = value
Assimilation rate µmol m^−2^ s^−1^			< 0,001
	Sifra	4,1979 ± 6,62^a^	
	Panamera	4,6764 ± 5,29^a^	
	Mondial	9,3259 ± 5,42^a^	
	Valor	9,5303 ± 3,90^a^	
	Tyson	14,2064 ± 7,74^ab^	
Transpiration rate mmol m^−2^ s^−1^			< 0,001
	Sifra	4,3866 ± 2,75^a^	
	Panamera	5,143 ± 3,18^a^	
	Tyson	5,1898 ± 1,91^a^	
	Mondial	7,2386 ± 1,79^ab^	
	Valor	9,3514 ± 4,91^b^	
Ambient (to leaf) CO2 µmol mol^−1^			< 0,001
	Tyson	775,7274 ± 12,45^b^	
	Valor	780,2541 ± 8,36^a^	
	Mondial	781,7653 ± 8,62^ab^	
	Panamera	789,9419 ± 9,70b^a^	
	Sifra	791,1256 ± 11,40^ac^	
Intercellular CO_2_ µmol mol^−1^			0,01
	Valor	618,9689 ± 32,08^a^	
	Mondial	690,3615 ± 111,96^a^	
	Tyson	677,3881 ± 102,07^a^	
	Panamera	682,8309 ± 91,58^a^	
	Sifra	905,0477 ± 519,13^ab^	
Stomatal conductance to water vapour mol m^−2^ s^−1^			< 0,001
	Panamera	0,1498 ± 0,13^a^	
	Sifra	0,1614 ± 0,11^a^	
	Valor	0,2671 ± 0,13^a^	
	Mondial	0,3368 ± 0,09^a^	
	Tyson	0,5452 ± 0,36^b^	
Total conductance to CO2 mol m^−2^ s^−1^			< 0,001
	Panamera	0,0912 ± 0,07^a^	
	Sifra	0,0987 ± 0,06^a^	
	Valor	0,1621 ± 0,07^a^	
	Tyson	0,3171 ± 0,19^c^	
	Mondial	0,2033 ± 0,05^ab^	

Means followed by different letters in columns indicate significant differences among treatments (Tukey’s test, *p* < 0.05)

*Phthorimaea absoluta* directly feeds on leaves, which reduces the overall surface area available for photosynthesis and decreases the plant’s assimilation rate. This difference was found to be significant between cultivars (F (4, 75) = 7.62, *p* < 0.001). The reduced leaf surface area, severe defoliation, and changes in ambient (F (4,75) = 6.61, *p* < 0.001) conditions around the plants also affected the plant’s ability to transpire. The difference in transpiration rate between the cultivars was found to be significantly different (F (4, 75) = 6.73, *p* < 0.001). *Phthorimaea absoluta* also affected other important component of photosynthesis, including intercellular carbon dioxide (F(4, 75) = 3.20, *p* < 0.001), stomatal conductance to water vapour (F(4, 75) = 10.77, *p* < 0.001), and total conductance to carbon dioxide (F (4, 75) = 11.21, *p* < 0.001).

### Pearson correlation analysis between morphological and physiological parameters

The Pearson correlation coefficients and significance levels between the number of leaves per plant, plant height, tuber weight (g), tuber quantity, transpiration rate mmol m^−2^ s^−1^, assimilation rate µmol m^−2^ s^−1^, ambient (to leaf) CO_2_ µmol mol^−1^, intercellular CO_2_ µmol mol^−1^, stomatal conductance to water vapour mol m^−2^ s^−1^, and total conductance to CO_2_ mol m^−2^ s^−1^ are displayed in Table 4. The tables show the Pearson correlation coefficients between variables and their significance levels. The following correlations were observed:

**Table 4:**
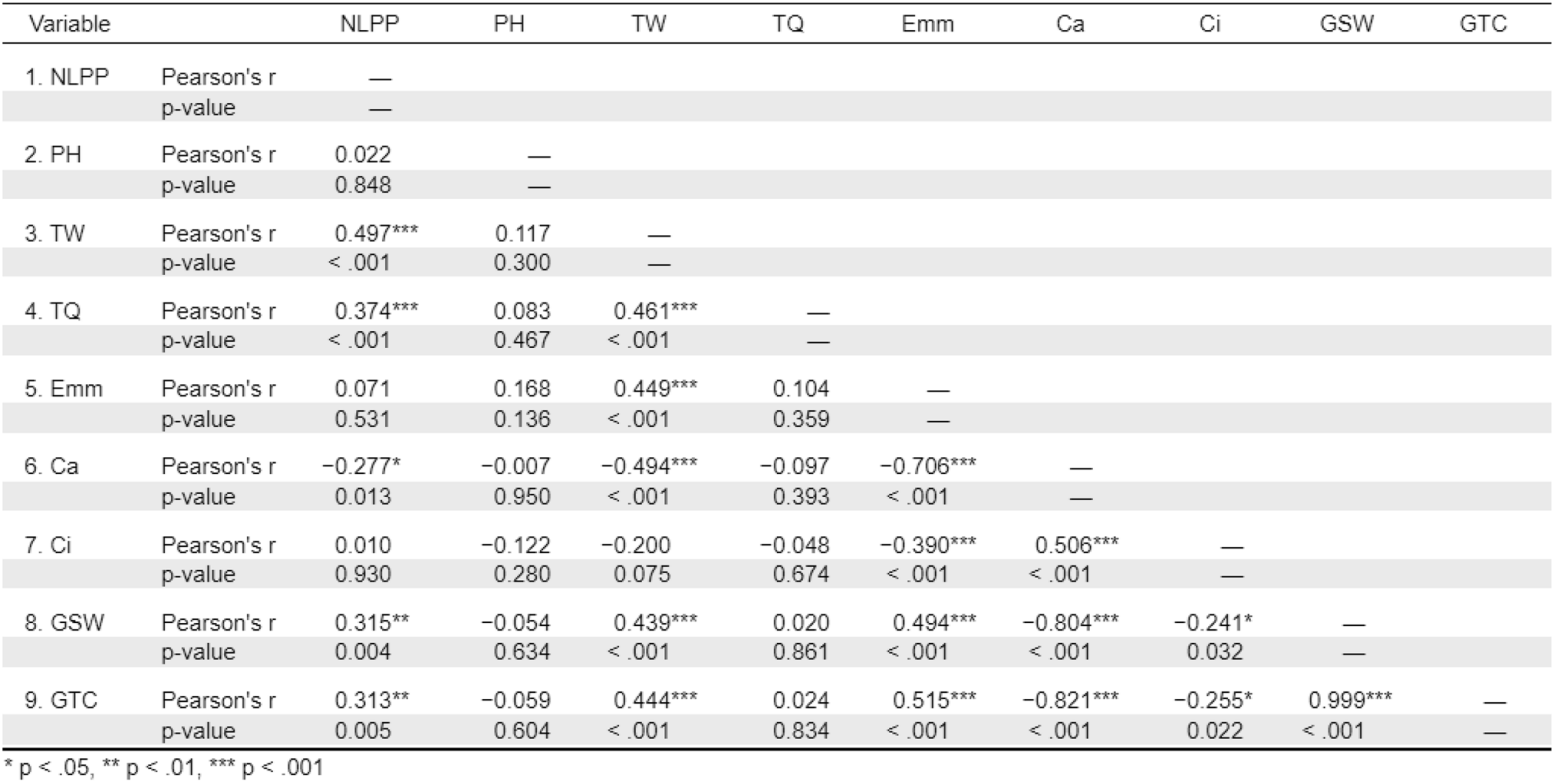
Person correlation analysis heat map of morphological and gas exchange parameters. NLPP = Number of leaves per plant, PH = Plant height (cm), TB = Tuber weight (g), TQ = Tuber quantity, Emm = Transpiration rate mmol m^−2^ s^−1^, A = Assimilation rate µmol m^−2^ s^−1^, Ca = Ambient (to leaf) CO_2_ µmol mol^−1^, Ci = Intercellular CO_2_ µmol mol^−1^, GSW = Stomatal conductance to water vapour mol m^−2^ s^−1^, and GTC = Total conductance to CO_2_ mol m^−2^ s^−1^

The number of leaves per plant positively correlated with assimilation rate (A), stomatal conductance to water vapour (gsw), total conductance to CO_2_ (gtc), weight, and tuber quantity. Tuber weight (g) positively correlated with several variables, including transpiration rate (Emm), assimilation rate (A), stomatal conductance to water vapour (gsw), total conductance to CO_2_ (gtc), number of leaves per plant, and tuber quantity. Transpiration rate (Emm) negatively correlated with ambient CO_2_ (Ca) and intercellular CO_2_ (Ci), but positively correlated with stomatal conductance to water vapour (gsw) and total conductance to CO_2_ (gtc). Ambient CO_2_ (Ca) was negatively correlated with transpiration rate (Emm), assimilation rate (A), stomatal conductance to water vapour (gsw), and total conductance to CO_2_ (gtc). Intercellular CO_2_ (Ci) was negatively correlated with transpiration rate (Emm), assimilation rate (A), stomatal conductance to water vapour (gsw), and total conductance to CO_2_ (gtc).

These results suggest that physiological performance (particularly gas exchange traits such as assimilation rate, stomatal conductance, and total conductance) plays a critical role in supporting plant morphology such as leaf number and plant height as well as yield components like tuber weight and quantity. The strong positive correlations among these variables indicate that plants with higher photosynthetic activity and conductance tend to produce more and larger tubers. Conversely, the negative correlations of ambient and intercellular CO₂ with key physiological traits reflect stress responses, likely due to pest pressure, which compromise photosynthesis and transpiration efficiency. Therefore, infestation may suppress gas exchange processes, indirectly reducing biomass accumulation and tuber production.

### Nutrient assimilation

Nutrient assimilation was examined in the most susceptible cultivar (Sifra), and the most tolerant cultivar (Valor). The analysis included phosphorus, potassium, calcium, magnesium, sodium, iron, copper, zinc, manganese, boron, and aluminium. However, only the nutrients that showed significant differences are reported, based on an independent t test with *p* < 0.05. The findings indicate consistent differences in nutrient assimilation between the tolerant cultivar, (Valor), and the susceptible cultivar, (Sifra), across all measured elements. The most notable variations were found in the profiles of macronutrients and micronutrients, with Valor exhibiting a superior capacity for accumulation.

**Fig. 4:**
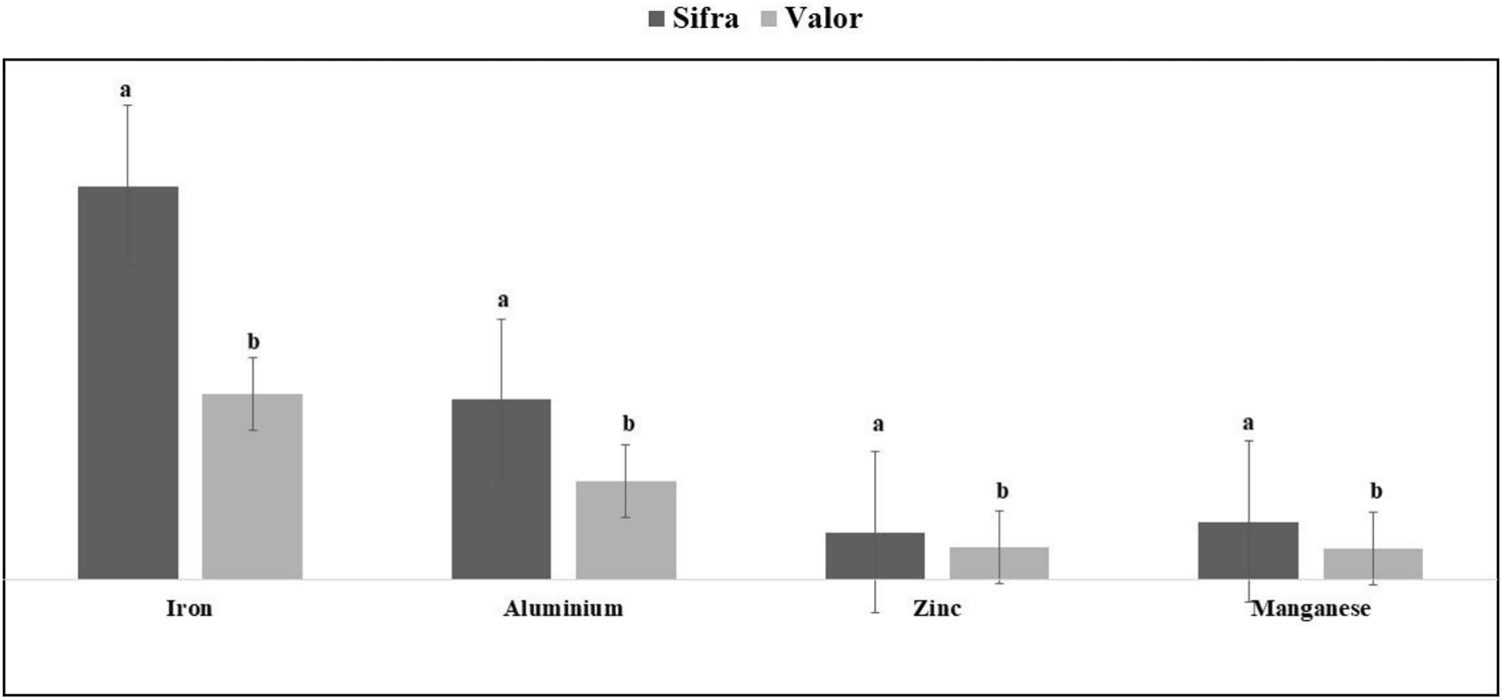
Nutrient assimilation between Valor (tolerant) and Sifra (susceptible) infested plants 45 days after infestation. Means followed by different letters in columns indicate significant differences among treatments (Mean ± SE)

Iron exhibited the most substantial difference between cultivars, with the tolerant cultivar (Valor) accumulating significantly higher levels of iron (34.0 ± 31.49) compared to the susceptible cultivar (Sifra) (16.02 ± 4.46), *p* < 0.03. The aluminium concentrations showed the second-largest disparity, with Valor maintaining notably higher levels (15.60 ± 11.40) than Sifra (8.06 ± 2.11), *p* < 0.01.The zinc levels were significantly higher in Valor (4.08 ± 3.98) than Sifra (2.73 ± 0.69), *p* < 0.001. The manganese concentrations in Valor (3.22 ± 2.24) exceeded those in Sifra (2.23 ± 0.86), *p* < 0.03 (Fig. 4).

## Discussion

Five widely cultivated South African potato varieties were examined to evaluate their response to *Phthorimaea absoluta* by analysing morphological parameters, gas exchange, and nutrient assimilation. While *P. absoluta* developed on and fed upon all the cultivars under study, there were notable differences in foliar damage and other assessed aspects, including morphology, gas exchange, and nutrient assimilation. Among the five cultivars, Sifra was the most susceptible, while Valor showed the highest tolerance, and the remaining three cultivars exhibiting intermediate responses.

Based on the plant scoring and growth parameters, the cultivars were ranked from most to least damaged as follows: Sifra, Tyson, Mondial, Panamera, and Valor. These findings align with previous research on varying responses of different tomato cultivars to *P. absoluta*, where some exhibited tolerance and others showed susceptibility (Sohrabi et al. 2016; Azadi et al. 2018).

The plant scoring revealed that Sifra had the highest number of mines per plant, followed by Tyson, Mondial, Panamera, and Valor (*p* < 0.001), with the number of leaves damaged and overall plant damage following the same pattern (*p* < 0.001). In all the cultivars except Panamera, infested plants were taller than uninfested plants (*p* < 0.001). The increased height of infested plants, except for Panamera, suggests a stress-induced shade avoidance response. In this response, herbivory reduces leaf area and alters the canopy's light quality, such as by decreasing the red/far-red light ratio, which mimics signals of competition from neighbouring plants (Pierik and De Wit 2014; Franklin 2008). This process triggers auxin-mediated internode elongation (Carriedo et al. 2016) to enhance light capture. However, herbivory-induced jasmonates simultaneously antagonise gibberellins signalling (Machado et al. 2017), limiting sugar allocation to tubers and restricting compensatory growth. The lack of height increase in Panamera likely reflects either a cultivar-specific attenuation of shade avoidance response (Carriedo et al. 2016) or a dominant jasmonate gibberellin antagonism that overrides elongation (Machado et al. 2017). The increased height observed in infested plants may reflect compensatory growth mechanisms, as described by McNaughton (1983). Herbivory can trigger resource reallocation or hormonal shifts that promote stem elongation, even when other fitness traits are compromised (Ramula et al. 2019).

While there was no significant difference in the number of potato tubers between cultivars (*p* > 0.32), there were notable differences in tuber weight (*P* < 0.01). This suggests that the infested plants produced several potato tubers compared to the uninfested plants, but the uninfested plants produced potato tubers with larger sizes. This suggests a trade-off between the number and size of tubers, where pest-stressed plants may produce more but smaller tubers. While this might maintain or even increase the total tuber count, the size reduction could lower market value and farmer profitability due to consumer preference for larger tubers. This aligns with the concept of plant tolerance, where infested plants prioritise the quantity of tubers over their size to ensure reproductive success under stress. In contrast, the non-infested plants allocate resources more efficiently, resulting in fewer but larger tubers. The trade-off between the number and size of tubers indicates a strategy of resource reallocation in response to herbivory, emphasising the adaptive nature of plant tolerance mechanisms (Koch et al. 2016).

The presence of pests on the plant can impair the plant’s physiological functions (Koch et al. 2016). *Phthorimaea absoluta* feeding may have damaged plant tissue, interfering with gas exchange and consequently reducing assimilation rates (*p* < 0.001). This finding is consistent with the findings of Zangerl et al. (2002), who demonstrated that pests affected plant biomass and photosynthesis.

*Phthorimaea absoluta* feeding behaviour reduces the leaf surface area accessible for transpiration (*p* < 0.001) and disrupts water balance, leading to lower transpiration rates in infested plants. The reduced leaf surface area, severe defoliation, and changes in ambient conditions (*p* < 0.001) also affected the plants’ ability to transpire, resulting in higher ambient temperatures around infested plants. Similar reduced transpiration due to pest infestation was observed by Shannag and Freihat (2009) on Solanaceae crop cucumber and Lamp et al. (2011) on potatoes infested with leafhopper. Increased transpiration from infested plants could exacerbate water stress, resulting in a decrease in plant productivity (Shannag and Freihat 2009). Infested plants showed lower stomatal conductance (*p* < 0.01), aligning with the findings of Hoback et al. (2015), who noted that pests such as Colorado potato beetle could influence stomatal conductance directly by inducing closure or opening.

Pearson correlation analysis revealed significant relationships between key growth, physiological traits and yield components in potato cultivars under stress from *P. absoluta*. The number of leaves per plant was positively correlated with the photosynthetic assimilation rate, stomatal conductance to water vapour, total conductance to carbon dioxide, tuber weight, and tuber quantity. This indicates the critical role of leaf health in maintaining optimal photosynthetic activity and overall plant performance (Saeed et al. 2007; Khayatnezhad et al., 2009). Tuber weight also exhibited positive correlations with transpiration rate, photosynthetic assimilation rate, stomatal conductance to water vapour, total conductance to carbon dioxide, number of leaves, and tuber quantity. This further emphasises that tuber weight is a strong indicator of both physiological function and yield potential (Zhang et al. 2021).

The transpiration rate was negatively correlated with ambient and intercellular carbon dioxide concentration but positively correlated with stomatal conductance to water vapour and total conductance to carbon dioxide, suggesting that stomatal regulation plays a significant role in managing water loss and carbon uptake under pest-induced stress (Saeed et al. 2007). Both ambient and intercellular carbon dioxide concentrations showed negative correlations with transpiration rate, photosynthetic assimilation rate, stomatal conductance to water vapour, and total conductance to carbon dioxide, highlighting the crucial role of carbon dioxide availability in optimising photosynthesis and mitigating stress impacts (Zhang et al. 2021). These findings align with previous studies that emphasised the importance of morphological traits, such as leaf number and tuber weight, for enhancing cultivar tolerance to biotic stress and ensuring high yields (Shubha and Singh 2018).

Nutrients play a significant role in plant stress response (Mahiwal and Panday, 2022). Our study demonstrates that Valor, the most tolerant cultivar, consistently showed higher nutrient assimilation across all the measured elements than Sifra, the most susceptible cultivar. The differences in nutrient profiles between these cultivars suggest that these elements may play a crucial role in defence mechanisms against *P. absoluta*. Valor also had a more enriched nutrient profile than Sifra, particularly in terms of iron, zinc, manganese, and aluminium. These micronutrients play essential roles in the activation of tolerance induction. Higher levels of iron, zinc, and manganese in Valor likely enhance defence responses. Iron serves as a cofactor for peroxidases and other enzymes involved in lignin and phenolic synthesis, contributing to a localised reactive oxygen species response that limits the spread of the pest (Dordas 2008; Trapet et al. 2021). Zinc activates antioxidant enzymes, such as superoxide dismutase, and supports defence signalling through salicylic and jasmonic acid pathways (Cabot et al. 2019; Bastakoti 2023). Manganese acts as a cofactor for enzymes involved in phenylpropanoid and lignin production, which strengthens cell walls and reduces tissue palatability to herbivores (Dordas 2008).

Valor tolerance is also backed by research from Dordas (2008) and Bastakoti (2023), who both demonstrated the promise of micronutrients like iron, zinc, manganese, and aluminium in enhancing plant defence. Iron levels were notably higher in Valor compared to Sifra (*p* < 0.03). This difference is particularly relevant as iron enhances resistance to biotic factors and specific diseases (Trapet et al. 2021; Dordas 2008). Aluminium concentrations were also higher in Valor than in Sifra (*p* < 0.01). This is significant because aluminium accumulation in leaf tissues forms both physical and chemical barriers that can deter insect herbivores from feeding. Additionally, aluminium can limit pathogen spread by inducing reactive oxygen species and activating defence pathways involving nitric oxide and salicylic acid (Ofoe et al. 2023).

Zinc concentrations were markedly higher in Valor and significantly lower in Sifra (*p* < 0.001). This difference is crucial as zinc is essential for activating key enzymes like superoxide dismutase that mitigate oxidative stress, a common response to pest attacks (Cabot et al. 2019; Bastakoti 2023). The oxidative burst, which often leading to rapid cell death, serves as a localised defence mechanism, limiting the spread of the pest and signalling further systemic responses across the plant. Zinc also primes plant defence by enhancing the activity of signalling pathways like salicylic and jasmonic acid, both crucial in mobilising systemic defence responses (Bastakoti 2023).

Valor’s manganese levels were significantly higher in comparison to Sifra (*p* < 0.03). The higher manganese content is essential for plants to fight against biotic factors (Dordas 2008), as it plays a vital role in lignin and phenol biosynthesis, photosynthesis, and other functions, including the inhibition of enzymes like aminopeptidase and pectin methylesterase.

These nutrient profile differences between Valor and Sifra suggest that balanced nutrition is crucial for resistance or susceptibility to biotic factors (Huber and Haneklaus 2007). The consistently higher nutrient levels in Valor compared to Sifra across all the elements show that optimal nutrient balance could be one of the ways to reduce dependence on chemical controls in pest management strategies (Bastakoti 2023).

The use of resistant cultivars in farming is important; it gives plants a fair chance to defend themselves against biotic factors such as *P, absoluta*. Most importantly, it reduces the dependence on chemicals as a pest management strategy. Given Valor’s strong performance across physiological and morphological parameters, it may be recommended for potato farming regions. This experiment was conducted in pots, which may have influenced plant growth and pest dynamics due to factors like limited root space, uniform soil conditions, and altered microclimates. These conditions can impact nutrient uptake, plant physiology, and pest behaviour. Therefore, the findings should be interpreted cautiously until they are validated in open-field conditions.

## Conclusion

This study identified Valor as the most tolerant cultivar and Sifra as the most susceptible cultivar. These findings suggest that incorporating Valor into IPM strategies could enhance pest resilience and decrease reliance on chemicals. Future research should confirm these traits through multi-environment field trials and investigate the molecular mechanisms that contribute to tolerance.

## Data Availability

Data are available on request.

## References

[CR1] Abstract of Agricultural Statistics (2022) Available online: http://www.dalrrd.gov.za Accessed on 15 July 2024.

[CR2] Ajibade II, Maduka NC, Murana KA (2017) The menace of *Tuta absoluta*: a review on the world spread. IOSR J Appl Geol Geophys 5(5):21–29

[CR3] Arnó J, Gabarra R (2010) Controlling *Tuta absoluta*, a new invasive pest in Europe. Train Integrated Pest Manag 5:1–8

[CR4] Azadi F, Rajabpour A, Lotfi Jalal Abadi A, Mahjoub M (2018) Resistance of tomato cultivars to *Tuta absoluta* (Lepidoptera: Gelechiidae) under field condition. J Crop Prot 7(1):87–92

[CR5] Bastakoti S (2023) Role of zinc in management of plant diseases: a review. Cogent Food Agric 9(1):2194483

[CR6] Biondi A, Guedes RNC, Wan FH, Desneux N (2018) Ecology, worldwide spread, and management of the invasive South American tomato pinworm, *Tuta absoluta*: past, present, and future. Annu Rev Entomol 63:239–258 28977774 10.1146/annurev-ento-031616-034933

[CR7] Cabot C, Martos S, Llugany M, Gallego B, Tolrà R, Poschenrieder C (2019) A role for zinc in plant defense against pathogens and herbivores. Front Plant Sci 10:1171 31649687 10.3389/fpls.2019.01171 PMC6794951

[CR8] Caparros R, Brostaux Y, Haubruge E, Verheggen FJ (2013) Propensity of the tomato leafminer, *Tuta absoluta* (Lepidoptera: Gelechiidae), to develop on four potato plant varieties. Am J Potato Res 90(3):255–260

[CR9] Carriedo LG, Maloof JN, Brady SM (2016) Molecular control of crop shade avoidance. Curr Opin Plant Biol 30:151–158 27016665 10.1016/j.pbi.2016.03.005

[CR10] Chhetri LB (2018) Tomato leafminer (*Tuta absoluta*), an emerging agricultural pest: control and management strategies: a review. World Sci News 114:30–43

[CR11] Desneux N, Han P, Mansour R, Arnó J, Brévault T, Campos MR, Chailleux A, Guedes RN, Karimi J, Konan KA, Lavoir AV (2022) Integrated pest management of *Tuta absoluta*: practical implementations across different world regions. J Pest Sci 1:1–23

[CR12] Dordas C (2008) Role of nutrients in controlling plant diseases in sustainable agriculture. A Rev Agron Sustain Dev 28:33–46

[CR13] Erdoğan P, Babaroğlu NE (2014) Life table of the tomato leaf miner, *Tuta absoluta* (Meyrick) (Lepidoptera: Gelechiidae). J Agric Fac Gaziosmanpaşa Univ 2:80–89

[CR14] European Plant Protection Organization (EPPO) (2005) Data sheets on quarantine pests: *Tuta absoluta*. EPPO Bull 35:434–435

[CR15] Franklin KA (2008) Shade avoidance. New Phytol 179(4):930–944 18537892 10.1111/j.1469-8137.2008.02507.x

[CR16] Galdino TVDS, Picanço MC, Ferreira DO, Silva GAR, De Souza TC, Silva GA (2015) Is the performance of a specialist herbivore affected by female choices and the adaptability of the offspring? PLoS ONE 10(11):e0143389 26600074 10.1371/journal.pone.0143389 PMC4658099

[CR17] Ghaderi S, Fathipour Y, Asgari S (2017) Susceptibility of seven selected tomato cultivars to *Tuta absoluta* (Lepidoptera: Gelechiidae): implications for its management. J Econ Entomol 110(2):421–429 28334083 10.1093/jee/tow275

[CR18] Gómez-Trejo LF, Hernández-Acosta E, Peralta-Sánchez MG (2021) N, P, K nutrition differentially affects the incidence and severity of the attack of pests and diseases in plants. Agro Productividad 14(5):121–125

[CR19] Hoback WW, De Freitas BA, Martinez CA, Higley LG, Fernandes OA (2015) Photosynthetic responses of potato to Colorado potato beetle injury and differences in injury between adult males and females. Entomol Exp Appl 157(2):181–187

[CR20] Huber DM, Haneklaus S (2007) Managing nutrition to control plant disease. Landbauforsch Volkenrode 57(4):313

[CR21] Khayatnezhad M, Shahriari R, Gholamin R, Jamaati-e-Somarin S, Zabihi-e-Mahmoodabad R (2011) Correlation and path analysis between yield and yield components in potato (*Solanum tubersum* L.). Middle-East J Sci Res 7(1):17–21

[CR22] Koch KG, Chapman K, Louis J, Heng-Moss T, Sarath G (2016) Plant tolerance: a unique approach to control hemipteran pests. Front Plant Sci 7:1363 27679643 10.3389/fpls.2016.01363 PMC5020058

[CR23] Lamp WO, Miranda D, Culler LE, Alexander LC (2011) Host suitability and gas exchange response of grapevines to potato leafhopper (Hemiptera: Cicadellidae). J Econ Entomol 104(4):1316–1322 21882698 10.1603/ec11064

[CR24] Machado RA, Baldwin IT, Erb M (2017) Herbivory-induced jasmonates constrain plant sugar accumulation and growth by antagonizing gibberellin signaling and not by promoting secondary metabolite production. New Phytol 215(2):803–812 28631319 10.1111/nph.14597

[CR25] Mahiwal S, Pandey GK (2022) Potassium: a vital nutrient mediating stress tolerance in plants. J Plant Biochem Biotechnol 31(4):705–719

[CR26] Mahlangu L, Sibisi P, Nofemela RS, Ngmenzuma T, Ntushelo K (2022) The differential effects of *Tuta absoluta* infestations on the physiological processes and growth of tomato, potato, and eggplant. InSects 13(8):754 36005379 10.3390/insects13080754 PMC9409810

[CR27] Mansour R, Brévault T, Chailleux A, Cherif A, Grissa-Lebdi K, Haddi K, Mohamed SA, Nofemela RS, Oke A, Sylla S, Tonnang HE (2018) Occurrence, biology, natural enemies and management of *Tuta absoluta* in Africa. Entomol Gen 38(2):83–112

[CR28] McNaughton SJ (1983) Compensatory plant growth as a response to herbivory. Oikos 1:329–336

[CR29] Neves AD, Oliveira RF, Parra JR (2006) A new concept for insect damage evaluation based on plant physiological variables. An Acad Bras Cienc 78:821–835 17143415 10.1590/s0001-37652006000400015

[CR30] Ofoe R, Thomas RH, Asiedu SK, Wang-Pruski G, Fofana B, Abbey L (2023) Aluminium in plant: benefits, toxicity and tolerance mechanisms. Front Plant Sci 13:1085998 36714730 10.3389/fpls.2022.1085998 PMC9880555

[CR31] Pereyra PC, Sánchez NE (2006) Effect of two solanaceous plants on developmental and population parameters of the tomato leaf miner, *Tuta absoluta* (Meyrick) (Lepidoptera: Gelechiidae). Neotrop Entomol 35:671–676 17144141 10.1590/s1519-566x2006000500016

[CR32] Pierik R, De Wit M (2014) Shade avoidance: phytochrome signalling and other aboveground neighbour detection cues. J Exp Bot 65(11):2815–2824 24323503 10.1093/jxb/ert389

[CR33] Potato South Africa (PSA) (2017) Potato Industry Report. https://potatoes.co.za/# Accessed on 23 Aug 2024.

[CR34] Ramula S, Paige KN, Lennartsson T, Tuomi J (2019) Overcompensation: a 30-year perspective. Ecology 100(5):e02667 30913306 10.1002/ecy.2667 PMC6850278

[CR35] Resende JTVD, Maluf WR, Faria MV, Pfann AZ, Nascimento IRD (2006) Acylsugars in tomato leaflets confer resistance to the South American tomato pinworm, *Tuta absoluta* Meyr. Sci Agric 63:20–25

[CR36] Rwomushana I, Beale T, Chipabika G, Day R, Gonzalez-Moreno P, Lamontagne-Godwin J, Makale F, Pratt C and Tambo J (2019) Tomato leaf miner (*Tuta absoluta*): Impacts and coping strategies for Africa. CABI (Center for Agriculture and Bioscience International) Working Paper 12, pp. 1–56.

[CR37] Saeed IA, MacGuidwin AE, Rouse DI, Malek CA (2007) Field study on the influence of *Verticillium dahliae* and *Pratylenchus penetrans* on gas exchange of potato. Plant Dis 91(12):1531–1535 30780611 10.1094/PDIS-91-12-1531

[CR38] Shannag H, Freihat N (2009) Gas exchange of cucumber, *Cucumis sativus* L., impaired by tobacco whitefly, *Bemisia tabaci* (Gennadius) (Hemiptera: Aleyrodidae). Jordan J Agric Sci 5(3):295–305

[CR39] Shiberu T, Getu E (2017) Determination of oviposition preference and infestation level of *Tuta absoluta* on major Solanaceae crops under glasshouse conditions in Ethiopia. Adv Crop Sci Technol 5(6):1–7

[CR40] Shubha KS, Singh DS (2018) Selection of yield-associated morphological and biochemical traits using correlation and path coefficient analysis in potato (*Solanum tuberosum* L.) in the foothills of north-western Himalayas. Potato Res 61:273–281

[CR41] Sohrabi F, Nooryazdan H, Gharati B, Saeidi Z (2016) Evaluation of ten tomato cultivars for resistance against tomato leaf miner, *Tuta absoluta* (Meyrick) (Lepidoptera: Gelechiidae) under field infestation conditions. Entomol Gen 36(2):163–175

[CR42] Sperdouli I, Andreadis S, Moustaka J, Panteris E, Tsaballa A, Moustakas M (2021) Changes in light energy utilization in photosystem II and reactive oxygen species generation in potato leaves by the pinworm *Tuta absoluta*. Molecules 26:2984 34069787 10.3390/molecules26102984 PMC8157303

[CR43] Sprenger H, Erban A, Seddig S, Rudack K, Thalhammer A, Le MQ, Walther D, Zuther E, Köhl KI, Kopka J, Hincha DK (2018) Metabolite and transcript markers for the prediction of potato drought tolerance. Plant Biotechnol J 16(4):939–950 28929574 10.1111/pbi.12840 PMC5866952

[CR44] Stenberg JA (2017) A conceptual framework for integrated pest management. Trends Plant Sci 22(9):759–769 28687452 10.1016/j.tplants.2017.06.010

[CR45] Tekinsoy M, Bakhsh A, Çalışkan ME (2024) Expression of Jaburetox 2-Ec in potato encodes resistance against *Tuta absoluta* Meyrick (Lepidoptera: Gelechiidae). Potato Res 68:1477–1490

[CR46] Trapet PL, Verbon EH, Bosma RR, Voordendag K, Van Pelt JA, Pieterse CM (2021) Mechanisms underlying iron deficiency-induced resistance against pathogens with different lifestyles. J Exp Bot 72(6):2231–2241 33188427 10.1093/jxb/eraa535

[CR47] Uzun F, Birgucu AK, Karaca I (2015) Determination of oviposition preference of *Tuta absoluta* to tomato, pepper and eggplant. Asian J Agric Food Sci 3(5):569–578

[CR48] Visser D, Uys VM, Nieuwenhuis RJ, Pieterse W (2017) First records of the tomato leaf miner *Tuta absoluta* (Meyrick, 1917) (Lepidoptera: Gelechiidae) in South Africa. BioInvasions 6:301–305

[CR49] Yang Y, Wang Y, Du G, Wang W, Sun G, Chen B, Zhang L (2024) Bottom-up effects of various potato cultivars on the performance of *Tuta absoluta*. Entomol Gen 44(2):315–322

[CR50] Younes AA, Zohdy ZMN, Abul FH, Fathy R (2018) Preference and performance of the tomato leafminer, *Tuta absoluta* (lepidoptera – gelechiidae): towards three solanaceous host plant species. CPQ Microbiol 1(3):1–16

[CR51] Zangerl AR, Hamilton JG, Miller TJ, Crofts AR, Oxborough K, Berenbaum MR, De Lucia EH (2002) The impact of folivory on photosynthesis is greater than the sum of its holes. Proc Natl Acad Sci U S A 99(2):1088–1091 11792866 10.1073/pnas.022647099 PMC117434

[CR52] Zhang M, Yan J, Ali A, Gao Y (2021) Different performance of *Phthorimaea operculella* Zeller (Lepidoptera: Gelechiidae) among four potato tuber varieties under laboratory condition. InSects 12(7):580–591 34202396 10.3390/insects12070580 PMC8303611

